# Selective modulation of cellular voltage-dependent calcium channels by hyperbaric pressure—a suggested HPNS partial mechanism

**DOI:** 10.3389/fncel.2014.00136

**Published:** 2014-05-27

**Authors:** Ben Aviner, Gideon Gradwohl, Merav Mor Aviner, Shiri Levy, Yoram Grossman

**Affiliations:** ^1^Department of Physiology and Neurobiology, Ben Gurion University of the NegevBeer Sheva, Israel; ^2^Department of Physics, Jerusalem College of TechnologyJerusalem, Israel

**Keywords:** high pressure neurological syndrome, hyperbaric helium pressure, high pressure, voltage dependent calcium channels, synaptic transmission, conformational change, voltage dependency

## Abstract

Professional deep sea divers experience motor and cognitive impairment, known as High Pressure Neurological Syndrome (HPNS), when exposed to pressures of 100 msw (1.1 MPa) and above, considered to be the result of synaptic transmission alteration. Previous studies have indicated modulation of presynaptic Ca^2+^ currents at high pressure. We directly measured for the first time pressure effects on the currents of voltage dependent Ca^2+^ channels (VDCCs) expressed in *Xenopus* oocytes. Pressure selectivity augmented the current in Ca_V_1.2 and depressed it in Ca_V_3.2 channels. Pressure application also affected the channels' kinetics, such as Ʈ_Rise_, Ʈ_Decay_. Pressure modulation of VDCCs seems to play an important role in generation of HPNS signs and symptoms.

## Introduction

### HPNS

Every chemical reaction contributing to the continued existence of an organism is a potential target for pressure effects on biological processes. The major neurological problems associated with hyperbaric environments include N_2_ narcosis (inert-gas narcosis); O_2_ toxicity, which occurs due to increased oxidative stress (Allen et al., 2009); and HPNS (Halsey, 1982; Talpalar and Grossman, 2006). By controlling partial-pressures of absorbed tissue gases while under pressure, these neurological problems, excluding HPNS, can be alleviated and even eliminated, leading to the notion that HPNS occurs due to effects of pressure *per se* (Abraini, 1997; Bennett, 1997). Deep sea divers (approximately >100 m), as well as animals exposed to hyperbaric pressure (HP), may experience HPNS, which in humans includes dizziness, nausea, tremors, vision, and auditory disturbances, decrements in locomotor activity (Tarasiuk and Grossman, 1990; Darbin et al., 2000) and intellectual performance (Logue et al., 1986; Vaernes et al., 1988; Overman et al., 1989; Abraini, 1997; Steevens et al., 1999), confusion, changes in EEG and sleep disorders (Rostain et al., 1997), myoclonus (Darbin et al., 2000), convulsions, and loss of consciousness.

#### Synaptic transmission

Changes in synaptic transmission properties is a possible explanation for this constellation of signs and symptoms (for review see Daniels and Grossman, 2003). Indeed, it has previously been shown that synaptic transmission is suppressed by HP whether in vertebrate CNS synapse (Parmentier et al., 1981; Schleifstein-Attias et al., 1994), squid giant synapse (Parmentier et al., 1981), or crustacean neuromuscular junction (NMJ) (Campenot, 1975; Grossman and Kendig, 1990), either in excitatory (Grossman and Kendig, 1988; Golan et al., 1994) or inhibitory (Golan et al., 1994) synapses. Postsynaptic reduction in receptor sensitivity, observed in glycine receptors (Shelton et al., 1993), will exacerbate pressure-induced suppression in specific synapses (Daniels and Grossman, 2003). Various mechanisms for HP effects on synaptic transmission have been suggested over the past few decades, including modulation of ionotropic receptors activity (Heinemann et al., 1987; Shelton et al., 1993), decreased action potential (AP) amplitude (Aviner et al., 2013) and slowed kinetics (Grossman and Kendig, 1986; Etzion and Grossman, 1999), generally observed depression of neurotransmitter release (Parmentier et al., 1981; Ashford et al., 1982; Gilman et al., 1987; Etzion et al., 2008), decreased vesicle fusion (Ashford et al., 1982; Heinemann et al., 1987), and the reduction of Ca^2+^ currents (Talpalar and Grossman, 2003; Aviner et al., 2013). HP also causes a decrease in the frequency of spontaneous miniature excitatory postsynaptic potentials (Ashford et al., 1982), which may also suggest that [Ca^2+^]_i_ could be involved in manifesting these HP effects. Indeed, it was also found that HP mimics the effects of low [Ca^2+^]_o_ (Grossman and Kendig, 1990; Etzion et al., 2008), and in contrast, high [Ca^2+^]_o_ can antagonize HP effects (Golan and Grossman, 1992; Talpalar et al., 2010; Aviner et al., 2013). Taken together, we may postulate that one of the major mechanisms by which HP suppresses synaptic transmission is a depression of Ca^2+^ influx into the presynaptic terminal through voltage-dependent Ca^2+^ channels (VDCC), which is the natural trigger of this process. Indeed, Grossman and his colleagues have presented indirect (Grossman and Kendig, 1990; Grossman et al., 1991; Etzion and Grossman, 2000) and semi-direct (Aviner et al., 2013) evidence for this HP effect on voltage-dependent Ca^2+^ currents.

#### Pressure- and voltage-dependent ca^2+^ channels

Various VDCC types exist, characterized by their electrophysiological and pharmacological traits (Ca_V_1.1-4, Ca_V_2.1-3, Ca_V_3.1-3) and comprising the α_1_, α_2_δ, β, and γ subunits (Meir et al., 1999; Catterall, 2000). The major difference between the channels results from the variation in the α_1_ subunit, which holds the ion conducting pore, the voltage sensor, the channel gating section, and the known sites of channel regulation by second messengers, drugs, and toxins (Ertel et al., 2000). Evidence has accumulated, mostly indirectly, for HP effects on VDCC (Aviner et al., 2010). Pressure inhibited the brief reversal of swimming direction in the *Paramecium*, suggesting that an unclassified Ca^2+^ current that is associated with the reflex is impaired (Otter and Salmon, 1985). Depolarization-dependent Ca^2+^ influx into rat brain synaptosomes was depressed by pressure (Gilman et al., 1986). Membrane retrieval, another Ca^2+^-dependent process (Vogel et al., 1999; Wu et al., 2009), was also inhibited by pressure (Heidelberger et al., 2002).

Analysis of studies on crustacean neuromuscular synapses that examined the relationship between [Ca^2+^]_o_, EPSC amplitude, and facilitation (Grossman and Kendig, 1988, 1990; Golan and Grossman, 1992) suggests that pressure is acting to reduce Ca^2+^ influx, rather than to affect intracellular removal of Ca^2+^ or the release process.

To date, no attempt was made to directly measure isolated Ca^2+^ currents at pressure. The work detailed in this manuscript aims to do so for the first time.

Alteration in the properties of these channels at pressure can significantly influence one's cognition, sensual perception, and ability to control posture and muscle activity in a manifestation that could depict the HPNS.

## Methods

### Oocytes extraction and cRNA injection

Oocytes of a *Xenopus laevis* mature female frog were surgically extracted from its ovary and treated with 1.5 mg/ml collagenase for 30–60 min in order to remove connecting tissue. Suitable oocytes were sorted out by size, quality, and developmental stage (VI), and kept in NDE96 solution containing (in mM): 96 NaCl, 2 KCl, 1 MgCl_2_, 1 CaCl_2_, 2.5 sodium pyruvate; 50 μg/ml gentamycin; 5 HEPES pH 7.5.

cRNAs of the subunits of L or T type Ca^2+^ channels (LTCC or TTCC, respectively) were synthesized from rabbit cDNA by *in vitro* transcription with T7 or SP6 Amplicap High-Yield Message Maker Kit (Epicentre Technologies, Madison, WI). Oocytes were then injected with the specific cRNA mix (2.5 ng) encoding for the pertinent subunits to express LTCC or TTCC and were kept in an incubator for 4 days at 18°C in NDE96 solution. The following subunits were used: α_1C_+β_2A_+α_2_δ, comprising the LTCC Ca_V_1.2; and α_1H_+α_2_δ, comprising the TTCC Ca_V_3.2, whose gene code numbers are α_2_δ—M21948, β_2_—X64297, α_1C_—X15539, and α_1H_—AF051946.

### Electrophysiological recordings

Four days post-injection the oocytes were placed in a specially designed bath, and two-electrode voltage clamp (TEVC) experiments with 10 mV increments and 5–10 s interval between −70 and 40 mV were performed inside a compression chamber, utilizing an AXOCLAMP 2B amplifier (Molecular Devices, Axon Instruments, Inc., CA, USA), Master-8-cp Pulse Generator (A.M.P.I.), and AxoScope 9.2 software. While in the chamber, each oocyte was continuously perfused with a Ba^2+^ solution containing (in mM): 40 Ba(OH)_2_, 50 NaOH, 2 KOH, and 5 HEPES, titrated to pH 7.5 with methanesulfonic acid. Ba^2+^ was used as charge carrier, replacing the Ca^2+^ ions, in order to avoid Ca^2+^-dependent inactivation and the activation of Ca^2+^-activated Cl^−^ channels (Cl^−^_Ca_), known to be endogenously expressed in oocyte membrane (Miledi and Parker, 1984). The channels also have higher conductance to Ba^2+^ (Tsien et al., 1987), allowing measurement of minute currents that otherwise would have been unnoticed. The solution, saturated with air at atmospheric pressure, was introduced to the chamber by the use of a high pressure pump (Minipump, LDC Analytical Inc., Riviera Beach, FL, USA) at room temperature (24–25°C), at a rate of 1.5–2 ml/min. Temperature was constantly monitored throughout the experiments by the use of a thermistor submerged in the solution in the vicinity of the oocyte groove. Deviation of only ±0.5°C was allowed from the control temperature for later measurements.

**Figure 1:**
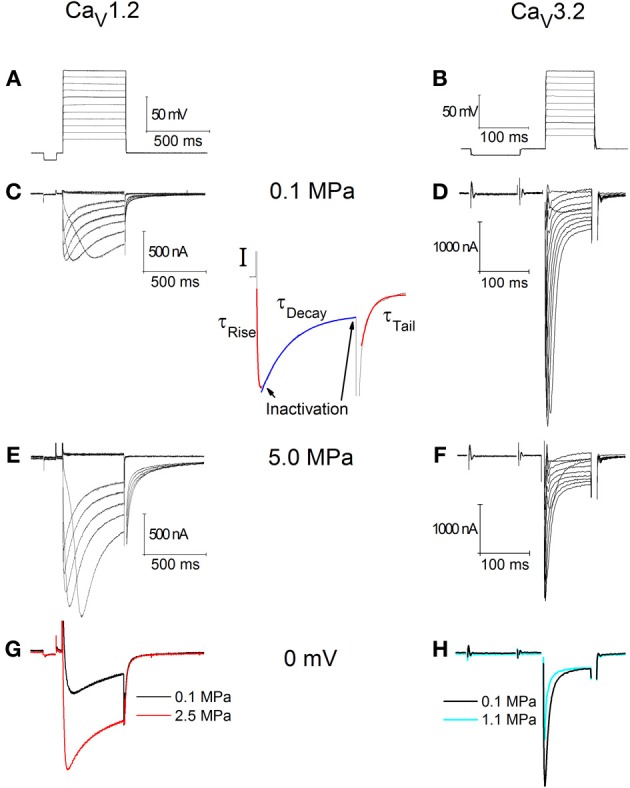
**Currents recorded in Ca_V_1.2 (C,E,G) and Ca_V_3.2 (D,F,H) channels. (A)** Depolarization steps for **(C,E)**. **(B)** Depolarization step for **(D,F)**. **(C,D)** Ba^2+^ currents at 0.1 MPa. **(E,F)** Ba^2+^ currents at 5.0 MPa. Note current increase in **(E)** at HP and decrease in **(F)**. **(G,H)** Superimposed single current traces under normo- and hyperbaric conditions, in response to identical depolarization to 0 mV. **(I)** Indication of the corresponding sections of the currents used to calculate the inactivation and kinetics parameters.

Typical recording traces are shown in Figures 1A–D. For the oocytes expressing Ca_V_1.2, holding potential was −70 mV (Figure 1A). The duration of each depolarizing step was 500 ms, which was preconditioned by a 100 ms hyperpolarizing step to −80 mV in order to release the VDCC from partial inactivation. The latter was also used to calculate and monitor the oocytes' instantaneous input resistance, which was accounted for at each recorded trace separately. For the oocytes expressing Ca_V_3.2 holding potential was −80 mV (Figure 1B). The duration of each depolarizing step was 100 ms, preconditioned by a 150 ms hyperpolarizing step to −90 mV, for similar reasons.

Every series of depolarizing pulses was used to construct an I-V curve and repeated at least three times in order to verify stability of the currents. Recorded traces with voltage fluctuation greater than 2 mV during depolarization were disregarded. We studied HP effects (examples in Figures 1C,D) on I-V curve, maximal currents, activation and inactivation functions, channel kinetics such as time to peak and time constants (Ʈ), and voltage dependency. Maximal currents were measured at the minimal point of the current curve (Figure 1I, left arrow). Inactivation (I/I_max_) was measured toward the end of the depolarizing step (Figure 1I, right arrow) in comparison to the measured maximal current (as above). A fit was calculated for each decaying section of the current in every recorded trace (Figure 1I, blue fit) according to a biexponential equation defining two time constants for decay:

$$\text{Fit}=-\text{A1exp}(\text{​}-t/{Ʈ}_{\text{Decay Fast}})-\text{A2exp}(\text{​}-t/{Ʈ}_{\text{Decay Slow}})+\text{C}.$$

For the rising phase and the tail currents a single exponential fit was performed (Figure 1I, red fits). All fits were calculated between the curves' normalized values of 0.1 and 0.9.

Activation volume (ΔV^‡^) was calculated for time constants of channel activation, inactivation, and deactivation under normobaric and hyperbaric conditions, following the known equation: ΔV^‡^ = RT(ƏlnƮ/ƏP)_T_.

### Truncated *K*^+^ channel

The current measurement using TEVC method was carried out for long durations and under HP conditions. Since it was our first attempt, verification of the recording stability was needed. As a first approximation, a truncated variant of a *Drosophila* K^+^ channel (KCNKØ) missing its carboxy-terminal tail (700 residues) was injected into oocytes (*n* = 4), causing a shift of their normal resting potential from about −40 to −50 mV to approximately −80 mV (Zilberberg et al., 2000). The membrane potential of these oocytes was then measured for 1–2 h and demonstrated stable resting potential of −80 ± 2 mV (not shown). This result showed that the setup measurements are accurate and do not drift throughout the duration of the experiment.

### Ca^2+^-activated Cl^−^ channel blocker

In an attempt to clarify whether Ba^2+^ currents measured in the VDCCs over-expressed in the oocyte membrane are subjected to artifacts caused by currents via endogenous channels, we performed a series of experiments in Ca_V_1.2 channels using 9-Anthracene carboxylic acid (9-AC), a pharmacological blocker of the Cl^−^_Ca_ (Boton et al., 1989). The use of Ca^2+^ chelators, e.g., BAPTA or EGTA, was not feasible due to their short time-span effectiveness in relation to the duration of our experiments (see Results, Time stability of HP effect). 9-AC was dissolved at 0.2–0.5 M in a 1 M solution of NaOH. This stock solution was then added to the physiological measurement solution to a final 2–4 mM concentration (Boton et al., 1989). Titration to pH 7.5 was then done using Methanesulfonic acid.

### Helium compression

After control measurement taken at 0.1 MPa, compression steps to 0.5, 1.0, 2.5, and 5.0 MPa were performed by compressed helium. Since the cDNA used to express these channels was of rodent origion, which is generally more resistant to HP than human, compressions greater than 1.0 MPa were also performed. Compression was done manually from a tank through a set of valves into the pressure chamber. Compression rate was approximately 0.25–0.5 MPa/min, and never exceeded 1.0 MPa/min. Helium was used instead of air due to its inert quality and the need to avoid known nitrogen narcosis and oxygen toxicity related effects (Dean et al.,, 2003). All pressure units are absolute.

### Statistical analysis

**Figure 2:**
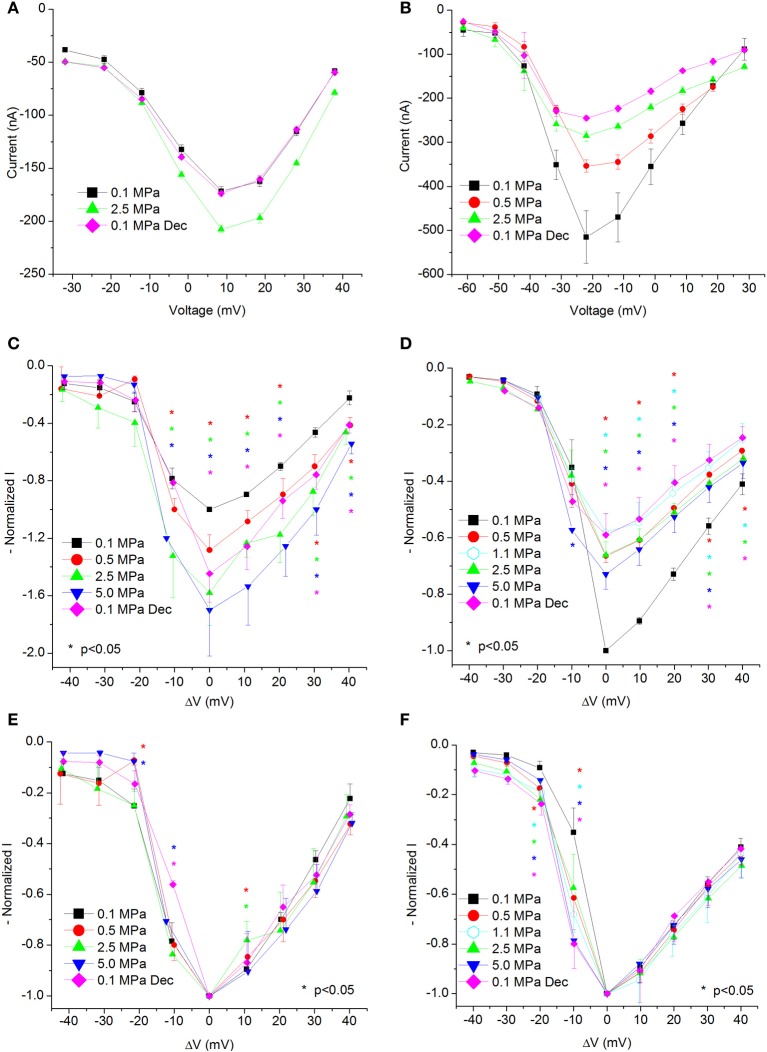
**I-V curves of maximal currents**. **(A,C,E)** Ca_V_1.2, **(B,D,F)** Ca_V_3.2 channels. **(A,B)** I-V curve of a single oocyte. **(C,D)** Pooled data from 9 to 17 **(C)** and 7 to 9 **(D)** oocytes exposed to 0.5–5.0 MPa pressure (color indicated). Holding potential is adjusted so that 0 indicates the potential at which maximal current is obtained (V_Imax_). **(E,F)** Normalized-to-max I-V curves. Each curve is normalized to its own maximal value and corresponds to its pertinent curve in **(C,D)**. Holding potential is adjusted as in **(C,D)**. Statistical significance for each point on the curve is indicated by corresponding color asterisks (*p* < 0.05). Dec indicates decompression.

**Figure 3:**
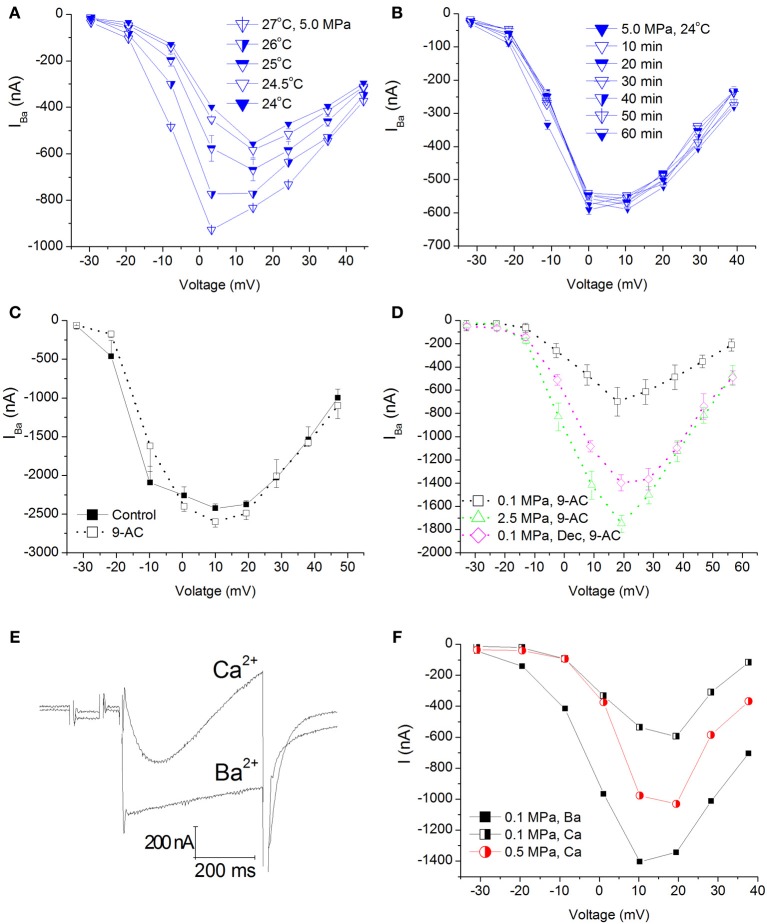
**Temperature, time, Cl^−^_Ca_ channel blocker, and Ca^2+^ ion control experiments in Ca_V_1.2 channel**. **(A)** I–V curves measured at 5.0 MPa repeatedly while the preparation's adiabatic temperature change is subsiding. **(B)** I-V curves measured in a different oocyte for 1 h at 5.0 MPa after control temperature was regained. **(C)** I-V curves measured in a solution containing 9-AC. **(D)** I-V curves of compression and decompression performed with 9-AC. The expected HP effect (augmentation) is evident. **(E)** Example of current traces recorded with Ca^2+^ or Ba^2+^ ions in the solution at the same depolarization to 0 mV. Note the stronger and faster inactivation with Ca^2+^. **(F)** Compression to 0.5 MPa when Ba^2+^ ions were replaced by Ca^2+^ ions. Replacement of ions caused a decrease of the currents at 0.1 MPa, but compression to 0.5 MPa still augmented the Ca^2+^ current in a similar manner to Ba^2+^ experiments.

**Figure 4:**
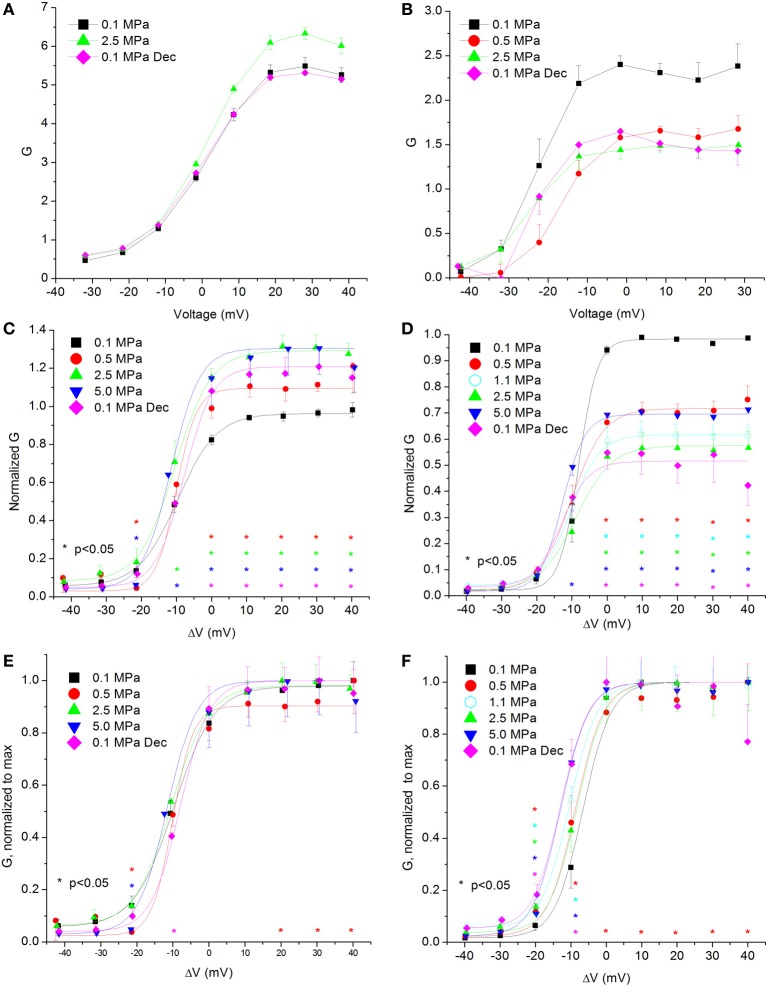
**Channels' conductance at various pressures. (A,C,E)** In Ca_V_1.2 and **(B,D,F)** in Ca_V_3.2 channels. **(A,B)** Conductance measured in a single oocyte. **(C,D)** Pooled data of the channels. **(E,F)** Normalized conductance: each curve is normalized to its own maximal value and corresponds to its pertinent curve in **(C,D)**. A Boltzmann fit was used in **(C–F)**. Pressures are color indicated. Statistical significance for each point on the curve is indicated by corresponding color asterisks. Holding potential is expressed as in Figure 2. Dec indicates decompression.

**Figure 5:**
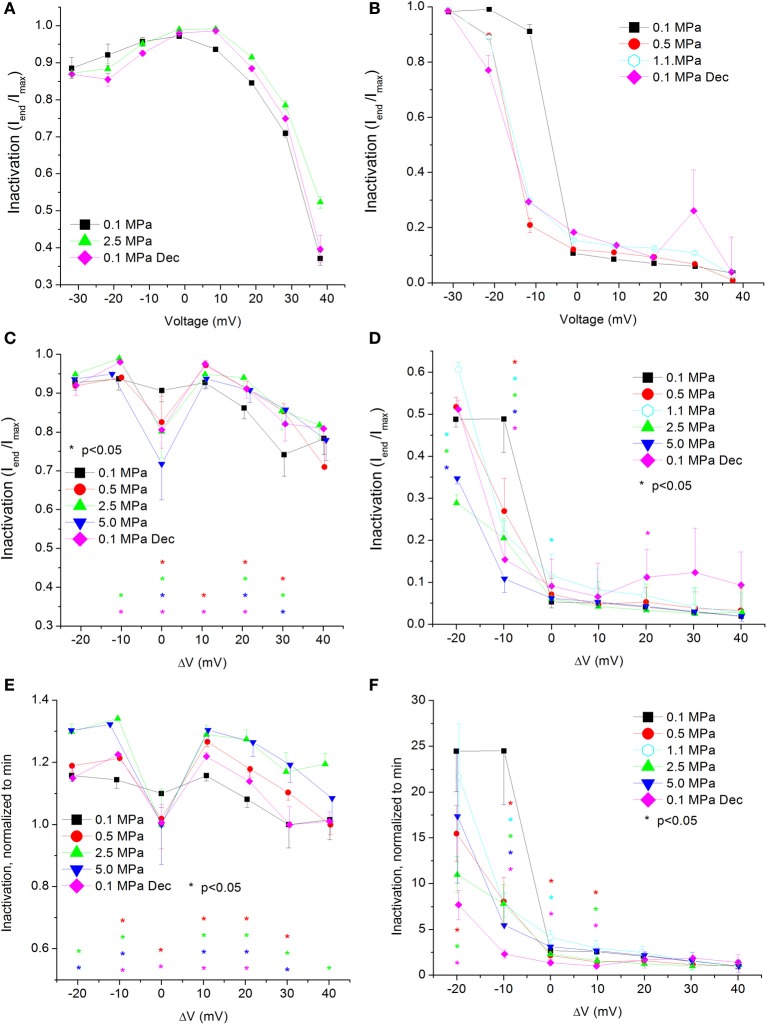
**Voltage- and time-dependent current inactivation (I_end_/I_max_) at various pressures. (A,C,E)** In Ca_V_1.2 and **(B,D,F)** in Ca_V_3.2 channels. **(A,B)** Inactivation measured in a single oocyte. **(C,D)** Pooled data of the channels. **(E,F)** Normalized inactivation: each curve is normalized to its own minimal value and corresponds to its pertinent curve in **(C,D)**. Pressures are color indicated. Statistical significance for each point on the curve is indicated by corresponding color asterisks. Holding potential is expressed as in Figure 2. Dec indicates decompression.

**Figure 6:**
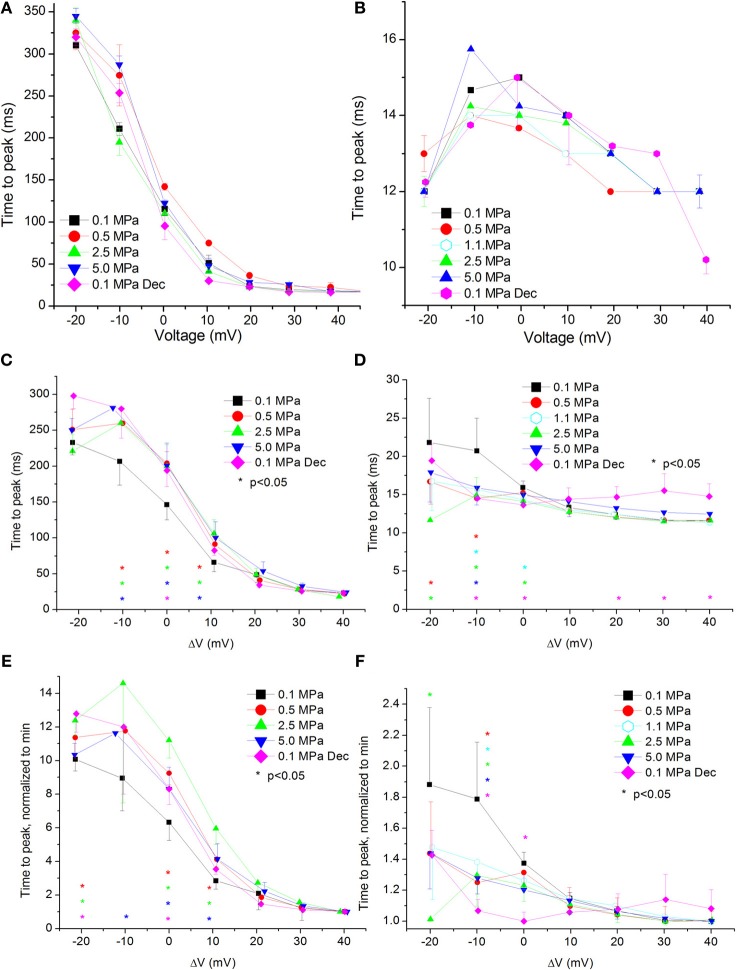
**Time to current peak (TTP, from stimulus onset) at various pressures. (A,C,E)** in Ca_V_1.2 and **(B,D,F)** in Ca_V_3.2 channels. **(A,B)** TTP measured in a single oocyte. **(C,D)**. Pooled data of the channels. **(E,F)** Normalized TTP: each curve is normalized to its own minimal value and corresponds to its pertinent curve in **(C,D)** Pressures are color indicated. Statistical significance for each point on the curve is indicated by corresponding color asterisks. Holding potential is expressed as in Figure 2. Dec indicates decompression.

**Figure 7:**
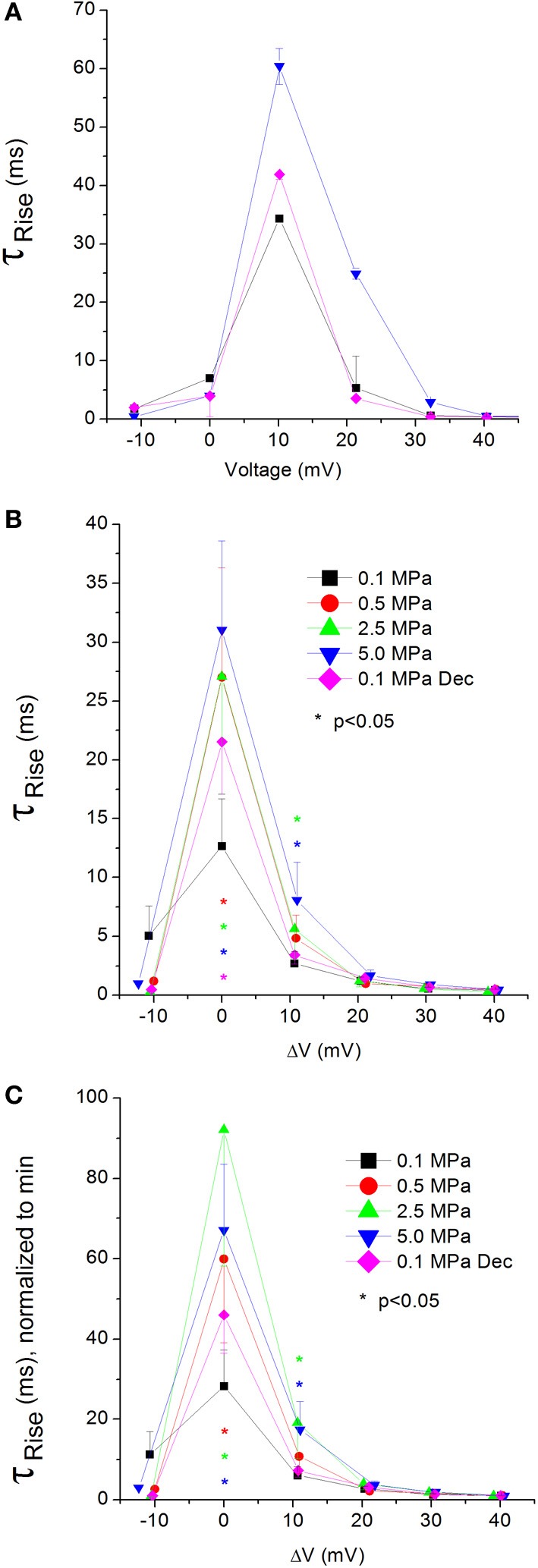
**Time constant of current activation (Ʈ_Rise_) in Ca_V_1.2 channel**. **(A)** Ʈ_Rise_ measured in a single oocyte. **(B)** Pooled data of Ʈ_Rise_ from 7 to 16 oocytes. **(C)** Normalized Ʈ_Rise_: each curve is normalized to its own minimal value and corresponds to its pertinent curve in **(B)**. Pressures are color indicated. Statistical significance for each point on the curve is indicated by corresponding color asterisks. Holding potential is expressed as in Figure 2. Dec indicates decompression.

**Figure 8:**
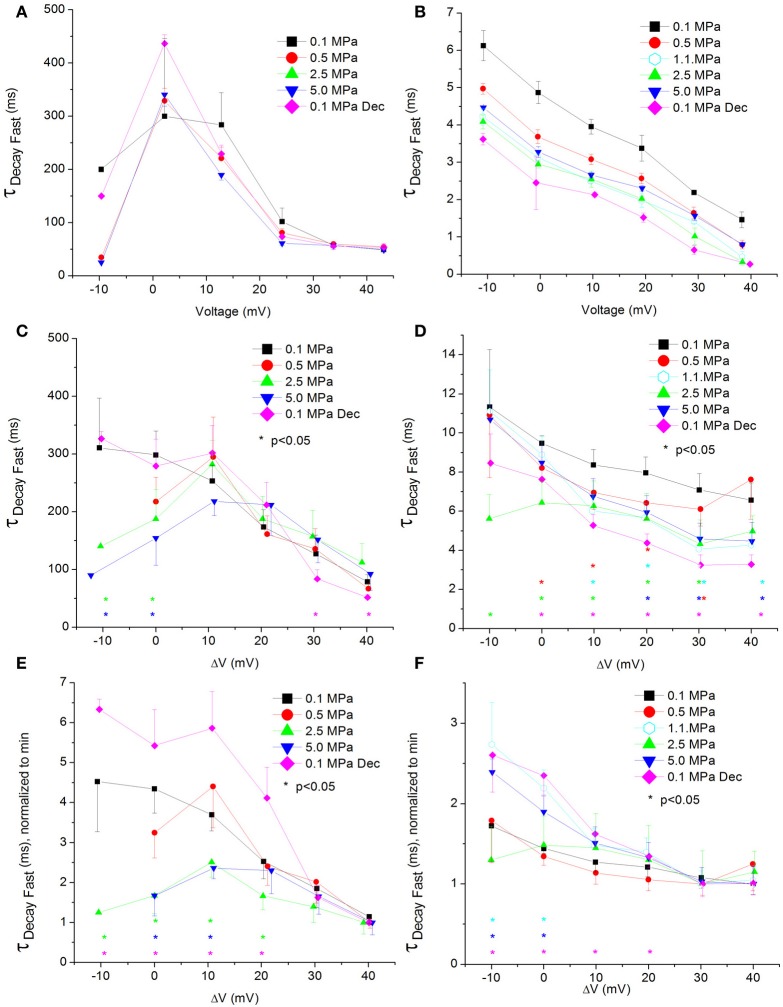
**Fast time constant of voltage- and time-dependent current inactivation (Ʈ_Decay Fast_). (A,C,E)** in Ca_V_1.2 and **(B,D,F)** in Ca_V_3.2 channels. **(A,B)** Ʈ_Decay Fast_ measured in a single oocyte. **(C,D)** Pooled data of the channels. **(E,F)** Normalized Ʈ_Decay Fast_: each curve is normalized to its own minimal value and corresponds to its pertinent curve in **(C,D)**. Pressures are color indicated. Statistical significance for each point on the curve is indicated by corresponding color asterisks. Holding potential is expressed as in Figure 2. Dec indicates decompression.

**Figure 9:**
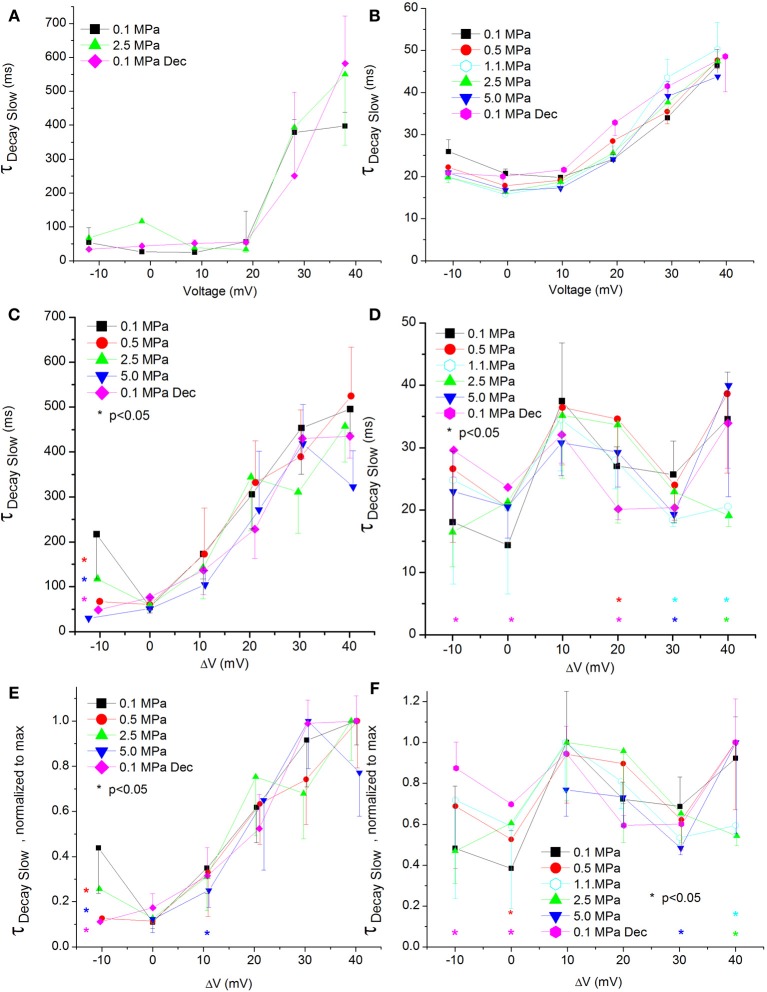
**Slow time constant of voltage- and time-dependent current inactivation (Ʈ_Decay Slow_). (A,C,E)** in Ca_V_1.2 and **(B,D,F)** in Ca_V_3.2 channels. **(A,B)** Ʈ_Decay Slow_ measured in a single oocyte. **(C,D)** Pooled data of the channels Ʈ_Decay Slow_. **(E,F)** Normalized Ʈ_Decay Slow_: each curve is normalized to its own maximal value and corresponds to its pertinent curve in **(C,D)**. Pressures are color indicated. Statistical significance for each point on the curve is indicated by corresponding color asterisks. Holding potential is expressed as in Figure 2. Dec indicates decompression.

The full set of parameters was calculated off-line for each recorded trace separately, considering the instantaneous input resistance and leak currents where appropriate, using a dedicated self-designed Matlab software program. The data were exported to Microsoft Excel software, by which averaging and binning were performed. Each oocyte was used as its own control, thus values were normalized to 0.1 MPa. When data from more than one oocyte were pooled, binning was performed relative to the voltage generating the maximal current in the I-V curve (V_Imax_); hence in figures representing these data (Figures 2 to 9C–F) the X-axis title is ΔV. Paired sample *t*-test was used to analyze the significance of the results.

## Results

### Increased current in Ca_V_1.2

Generally, HP was expected to suppress currents of VDCCs (see Introduction). Surprisingly, Ba^2+^ currents in Ca_V_1.2 were significantly increased in oocytes exposed to HP (0.5–5.0 MPa, see example in Figures 1E,G and 2A) in a dose-dependent manner, throughout the channels' voltage range of activity. Compression to 0.5, 2.5, and 5.0 MPa augmented the maximal currents by 28 ± 10, 58 ± 22, and 70 ± 32%, respectively (Figure 2C, Average ± SEM, *p* < 0.01, *n* = 9 − 17). Decompression back to 0.1 MPa only partially reversed the HP effect, leaving the maximal current 44 ± 19% increased. Neither the threshold voltage nor the depolarization (V_Imax_) that generated the largest currents (negative peak in IV curve) was affected.

Normalizing each IV curve under the various pressures to its own maximal (negative) peak (Figure 2E) shows almost identical curves, and therefore suggests that the channel's sensitivity to the membrane voltage did not alter as a result of the compression.

### Adiabatic temperature change and time stability

Elevation of ambient pressure in these experiments was achieved by gaseous (helium) compression; hence an adiabatic temperature rise always occurred (typically 0.5–2°C, but never more than 4°C), which was controlled by the rate of compression. Only measurements within 0.5°C or less of control temperature were taken under consideration. Therefore, it was necessary to wait for the chamber's atmosphere (bath fluid) to cool down (lasting 10–30 min), resulting in 1–2 h long experiments. Figure 3A shows IV curves in an oocyte expressing Ca_V_1.2 recorded at 5.0 MPa (post-compression) while cooling back to control temperature (24°C). As expected, elevated temperature (in addition to the HP) led to an increase in the currents. However, even after the control temperature was regained, the augmented current persisted. This strongly indicates that the temperature fluctuation cannot solely explain the increase in the channel's current and it should reflect a direct effect of HP (as also shown in Figure 2A). In order to verify that, and to rule out the possibility that the surprising augmentation of Ca_V_1.2 current at pressure is only the result of the adiabatic temperature change or a transient HP effect, currents were measured for 1–2 h post-compression (different experiments *n* = 2, Figure 3B). As can be seen, currents remained stable at HP for long durations after cooling back to control temperature, thus suggesting that the augmentation effect is stable and pressure induced.

### Eliminating Ca^2+^-activated Cl^−^ channels' current contamination

*Xenopus* oocytes are known to endogenously express the Ca^2+^-activated Cl^−^ channels. In order to avoid their activation (leading to inaccurate current measurement), we have used a Ba^2+^ solution with no added Ca^2+^ in our experiments. In order to avoid any current flowing through these channels in the unlikely event of their activation, the solution was also Cl^−^ free. Performing a TEVC experiment on naïve oocytes generated maximal currents in the 10–30 nA range (not shown), considered negligible compared to 1000 nA and more measured in oocytes expressing the Ca_V_1.2 channel. Nevertheless, we performed two sets of experiments aimed to verify that the theoretical Cl^−^_Ca_ current does not play a role in the HP-related effect, by adding 9-AC (a Ca^2+^-activated Cl^−^ channel blocker, see Methods) to the solution before or after compression (Figure 3C). Neither adding 9-AC pre-compression (Figure 3D) nor 9-AC added post-compression altered the HP-induced augmentation of Ca_V_1.2 current, indicating that the HP effect is real.

### Ba^2+^ −Ca^2+^ substitution

Since Ca^2+^ was substituted for Ba^2+^ due to the reasons mention in Methods, we wanted to verify that the HP effect is still valid when using the native Ca^2+^ ion. As expected, changing the bath solution to contain Ca^2+^ (using the Ca_V_1.2 channel) resulted in a decrease in the measured current and a faster and stronger inactivation (Figures 3E,F, black curves). Application of pressure augmented this current as well (Figure 3F, red curve).

### Decreased current in Ca_V_3.2

Ba^+^ currents in Ca_V_3.2 were significantly decreased at HP (0.5–5.0 MPa) in a dose-dependent manner (see example in Figures 1F,H and 2B), in contrast to the findings of Ca_V_1.2. Preliminary results showed that the HP effect did not change between 2.5 and 5.0 MPa; therefore another pressure step to 1.1 MPa was added in subsequent experiments in order to reveal the HP effect saturation. Neither the threshold voltage nor the V_Imax_ were affected. Compression to 0.5, 1.1, 2.5, and 5.0 MPa depressed the maximal currents by 33 ± 2, 42 ± 6, 34 ± 5, and 27 ± 5%, respectively (Figure 2D, Average ± SEM, *p* < 0.01, *n* = 4–9). Decompression to 0.1 MPa failed to recover the current, which remained depressed by 40 ± 7%. Normalizing each IV curve under the various pressure conditions to its own maximal (negative) peak (Figure 2F) reveals an increase of the currents in Ca_V_3.2 at sub-maximal membrane potentials (ΔV −10 mV), but not at V_Imax_ and above. We hypothesize that this might occur due to a greater probability for transition to the open state of the channel in this voltage range.

### Channel conductance

Calculating the channel conductance in relation to the membrane potential shows similar results to the general findings in the IV curves (see examples in Figures 4A,B). HP decreased the conductance in the Ca_V_3.2 channel (Figure 4D) and increased it in the Ca_V_1.2 channel (Figure 4C). On average, the change from threshold to maximal normalized response occurred within a 20 mV depolarization range for the Ca_V_3.2 channel and 30 mV for the Ca_V_1.2 channel. Normalizing each curve to its own maximal value (Figures 4E,F) reveals in the Ca_V_3.2 channel the same tendency seen in the normalized IV curve (Figure 2F) for greater conductance at HP in the ΔV −10 mV membrane potential, whereas the Ca_V_1.2 channel did not show such trait (Figure 4E). For depolarizations above V_Imax_ only compression to 0.5 MPa caused a consistent small reduction in normalized conductance in both channels.

### Current inactivation

The significant effect of HP on these channels manifested in the current and conductance parameters has led us to ask why pressure changes the total flux through them during supra-threshold depolarizations. A possible explanation could be that the channels' kinetics is affected by pressure. Previous findings have correlated HP to slowed inactivation in Na^+^ channels (Henderson and Gilbert, 1975; Conti et al., 1982b) and compared the effect of higher pressure to lower temperature (Grossman and Kendig, 1984, 1986). We therefore tested a few of the channels' kinetic properties. We first examined channel inactivation, measured as the ratio between the remaining fraction of the current at the end of the depolarizing voltage step and its maximal value (I_end_/I_max_; see examples in Figures 5A,B). The lack of Ca^2+^ ions in the experimental solution has eliminated the Ca^2+^-dependent inactivation and most likely guaranteed that only the voltage- and time-dependent inactivation will be measured. To verify this assumption, and in order to serve as a reference, a few experiments were conducted (*n* = 5) using a solution in which the Ba^2+^ was replaced with an equivalent concentration of Ca^2+^ (example in Figure 3E). As expected, in the presence of Ca^2+^ a stronger (2-fold) and faster (4-fold) inactivation was observed.

For the Ca_V_1.2 channel, inactivation was stronger at V_Imax_, but was weakened at stronger depolarizations (ΔV 10–40 mV, Figure 5C). Decompression did not relieve the HP effect. For the Ca_V_3.2 channel, the inactivation was stronger around the threshold voltage (ΔV −20 to −10 mV) at HP, but was weakened at V_Imax_ at lower HP (1.1 MPa).

Normalizing each curve to its own minimal value stresses the tendency for the strongest inactivation to occur in the Ca_V_1.2 channel at V_Imax_ at HP, whereas at normobaric pressure and post-decompression it occurs at the strongest depolarization, as expected for this VDCC (Figure 5E). For the Ca_V_3.2 channel, although the normalized curves converge at high depolarization, in most supra-threshold membrane potentials inactivation remained stronger at HP (Figure 5F).

### Currents time to peak

If the rate of activation of the VDCCs is affected by HP, that may change the time window for the ionic flow through them before the voltage- and time-dependent inactivation takes place. In case activation is as slow as inactivation, that will affect the I_max_ in each depolarization. We have therefore measured the time passing from the stimulating depolarizing step to the development of I_max_ (time to peak, TTP). Examples can be seen in Figures 6A,B.

For the Ca_V_1.2 channel the TTP was increased at HP near V_Imax_ (ΔV −10 to 10 mV, Figure 6C). Decompression did not relieve this effect.

For the Ca_V_3.2 channel TTP was decreased in the range between of ΔV −20 to 0 mV (Figure 6D). An increase of TTP was observed after decompression above V_Imax_.

Normalizing each TTP curve to its own minimal value (normally achieved at strong depolarization) showed the same tendencies: an increased TTP in the Ca_V_1.2 channel (Figure 6E) and a decreased TTP in the Ca_V_3.2 channel (Figure 6F).

### Currents Ʈ_Rise_

A complementary trait for the TTP in regard to the opening of the channel is the time constant of the rising phase of the current, Ʈ_Rise_. Slower TTP should mean greater Ʈ_Rise_, and vice versa. Indeed, in the Ca_V_1.2 HP caused an increase in the Ʈ_Rise_ values at V_Imax_ and 10 mV above (Figure 7A). Greater depolarizations led to convergence of Ʈ_Rise_ to control values (Figure 7B). Decompression only partially recovered Ʈ_Rise_, which remained elevated at V_Imax_. Normalizing each curve to its own minimal value demonstrates a similar rise in Ʈ_Rise_ at V_Imax_ and ΔV 10 mV (Figure 7C), which might support the notion that HP interferes with the opening mechanism of this channel, i.e., slows its kinetics. ΔV^‡^ of current activation for Ca_V_1.2 at V_Imax_ was calculated to be 454 ml/mole at 5.0 MPa.

Unfortunately, relatively large currents measured in the Ca_V_3.2 channel were accompanied by an artifact during their rising phase, which prevented accurate measurement of their Ʈ_Rise_.

### Currents fast Ʈ_Decay_

The changes in inactivation (I_end_/I_max_) found in both channels at HP could arise from its effect on their rate of decay, since I_end_ was measured here after a specific time and not under steady state conditions. The analysis revealed fast and slow time constants for the decay (see examples in Figures 8A,B, 9A,B), the first being shortened by stronger depolarizations and the latter being elongated.

For the Ca_V_1.2, HP caused a decrease in the fast Ʈ_Decay_ (Ʈ_Decay Fast_) at V_Imax_ and 10 mV below (Figure 8C). Stronger depolarizations (ΔV 10–40 mV) at HP did not change Ʈ_Decay Fast_, but after decompression its values were slightly smaller (ΔV 30–40 mV). In the rest of the depolarizing range, decompression fully recovered Ʈ_Decay Fast_. For the Ca_V_3.2, the ruling trend was a reduction of Ʈ_Decay Fast_ at HP, mainly from V_Imax_ and above (Figure 8D), while decompression did not relieve this effect. Normalizing each curve to its own minimal value showed for the Ca_V_1.2 channel a depression at 2.5 and 5.0 MPa, but not at 0.5 MPa (Figure 8E), while decompression increased the normalized Ʈ_Decay Fast_. In the Ca_V_3.2 channel at ΔV −10 to 0, compression to 1.1 and 5.0 MPa (but not to 0.5 and 2.5 MPa) increased the normalized Ʈ_Decay Fast_ (Figure 8F), which remained elevated after decompression in this activity range.

ΔV^‡^ of fast decay inactivation at V_Imax_ was calculated to be −332 and −55 ml/mole at 5.0 MPa for Ca_V_1.2 and Ca_V_3.2, respectively.

### Currents slow Ʈ_Decay_

The slow Ʈ_Decay_ (Ʈ_Decay Slow_) in both channels was increased by stronger depolarizations at 0.1 MPa (Figures 9A,B).

For the Ca_V_1.2 channel Ʈ_Decay Slow_ does not seem to be sensitive to HP, as no consistent significant difference was found in its values after compression or decompression (Figure 9C). For the Ca_V_3.2 channel Ʈ_Decay Slow_ was shortened only by strong depolarization (ΔV 30–40 mV) at HP, while decompression eliminated this effect (Figure 9D). The normalized curves of Ʈ_Decay Slow_ for both channels did not reveal a behavior different than that described above (Figures 9E,F).

ΔV^‡^ of slow decay inactivation at V_Imax_ was calculated to be −32 and 179 ml/mole at 5.0 MPa for Ca_V_1.2 and Ca_V_3.2, respectively.

### Currents Ʈ_Tail_

**Figure 10:**
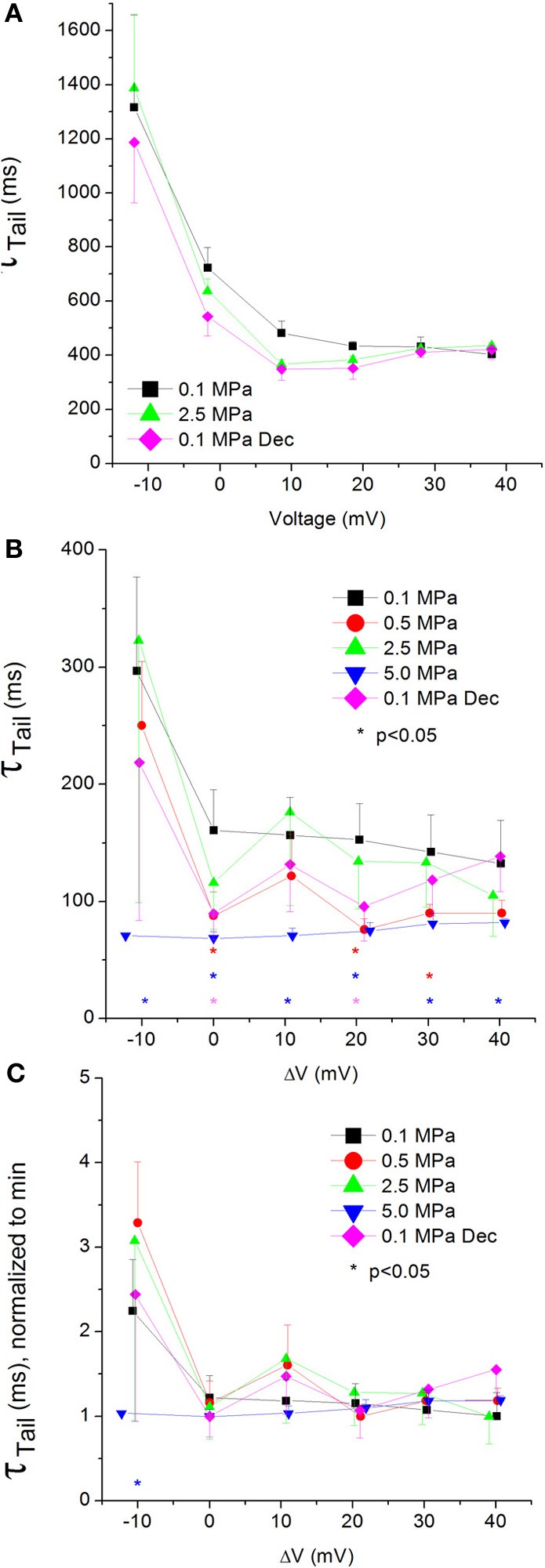
**Tail current time constant (Ʈ_Tail_) in Ca_V_1.2 channel. (A)** Ʈ_Tail_ measured in a single oocyte. **(B)** Pooled data of Ʈ_Tail_ from 7 to 16 oocytes. Only 5.0 MPa has affected (reduced) Ʈ_Tail_ throughout the channels' activity range. **(C)** Normalized Ʈ_Tail_: each curve is normalized to its own minimal value and corresponds to its pertinent curve in **(B)**. Pressures are color indicated. Statistical significance for each point on the curve is indicated by corresponding color asterisks. Holding potential is expressed as in Figure 2. Dec indicates decompression.

The tail current time constant (Ʈ_Tail_), representing the kinetics of the channels' deactivation, was shortened by increasing depolarization in the Ca_V_1.2 channel (see example in Figure 10A). This trend persisted with the application of HP, but at 5.0 MPa Ʈ_Tail_ remained minimal throughout the activity range of the channel (Figure 10B). Strong depolarization (ΔV 10–40 mV) at HP (0.5 and 5.0, but not 2.5 MPa) shortened Ʈ_Tail_. The normalized curves of Ʈ_Tail_ stress the absence of a significant slope at 5.0 MPa (Figure 10C). ΔV^‡^ of deactivation for Ca_V_1.2 at V_Imax_ was calculated to be −432 ml/mole at 5.0 MPa.

Unfortunately, the reciprocal artifact for the one interfering with measuring Ʈ_Rise_ in the Ca_V_3.2 channel also prevented an accurate measurement of Ʈ_Tail_.

## Discussion

### Establishing the methodology

The use of a truncated K^+^ channel proved the stability of measurements in our setup in these unusually long oocyte experiments, which were necessary due to adiabatic temperature fluctuations. Furthermore, there was no indication of long-term (1 h) effects of the fluctuating adiabatic temperature on barium currents once control temperature was restored (Figure 3B), suggesting that it cannot account for the much greater pressure-induced changes in maximal currents. Due to the relatively quick response of the preparation (Figure 3A), and in comparison to previous studies (Grossman and Kendig, 1984), it is likely that the time course of recovery from temperature transients is no longer than a few minutes, and therefore at the time of current measurements, when the ambient temperature is within 0.5°C deviation from control value, the membrane does not exhibit properties of temperature a few degrees higher or lower.

Additional support for this assumption arises from experiments in lobster (*Panulirus interruptus*) bifurcating axon, which showed that 10–15 min were sufficient to allow the responses to recover to the original levels and remain stable (Grossman and Kendig, 1986).

In the present experiments, cooling back to control temperature after compression steps lasted 12–15 min, with at least an additional 5–6 min required for verifying stability of currents. Thus, the total time of 17–21 min seems pertinent to allow the preparation to “relax” to its new pressure condition without temperature change interference. However, we cannot completely rule out the possibility that transient adiabatic temperature fluctuation contributes to the current change at pressure *per se*. In order to do so, conditions of compression need to allow a constant temperature during pressure changes, i.e., preventing or minimizing adiabatic temperature fluctuations. Only liquid compression can achieve this stipulation to a high degree, but unfortunately cannot be employed in our current setting. Nevertheless, Schmalwasser et al. (1998) have managed to design and operate a hydrostatic (oil) pressure chamber, in which adiabatic temperature fluctuation in compressions steps of 10 MPa was only 0.5°C or less, and demonstrated a reversible reduction of currents in the *Shaker* potassium channel mutant *Shaker* BD6-46 T449K. These results show that high pressure induced its effects on the channel even when temperature fluctuation was minimized, suggesting that our results were not distorted by the small temperature change.

The use of the 9-AC blocker both in normobaric and HP conditions strongly suggested that the endogenous Cl^−^_Ca_ currents are not of significant importance in our experiments (Figures 3C,D). Furthermore, the use of Ba^2+^ as a charge carrier proved to be efficient, as currents were stronger and the Ca^2+^ inactivation was eliminated (Figure 3E) as expected (Lyford et al., 2002).

Taken together, these control experiments show that the changes demonstrated after compression are indeed pressure related.

The HP-induced depression demonstrated in the Ca_V_3.2 channel further strengthened our findings in the Ca_V_1.2 channel, ruling out the possibility of it being a setup-related artifact.

### Currents' activation

#### Currents' amplitude

We show here, for the first time by direct measurement, that pressure effect can be selective: currents through Ca_V_1.2 are increased, whereas currents through the Ca_V_3.2 channel are depressed, at HP. It is noteworthy that the Ca_V_3.2 is more sensitive to pressure changes than the Ca_V_1.2 channel, as the HP effect is already saturated at 1.1 MPa. Furthermore, currents in the Ca_V_1.2 were partially recovered on return to atmospheric pressure, whereas in the Ca_V_3.2 currents did not recover. Although the Ca_V_3.2 channel is found in neuronal somata and much less in the nerve terminals, the findings demonstrate the possibility of selective pressure effects on other types of Ca^2+^ channels (P/Q, N, R) that are believed to be present at the presynaptic nerve terminals (Uchitel et al., 1992; Etzion and Grossman, 2000). Such a selectivity was already indirectly suggested in our experiments on frog motor nerve terminals (Aviner et al., 2013).

The effect on the Ca_V_1.2 is apparently in contrast to previous reports that suggested reduction in Ca^2+^ influx through Ca_V_2.2 (Grossman et al., 1991), and a lesser (Etzion and Grossman, 2000; Aviner et al., 2013) or no effect (Heinemann et al., 1987) in L-type VDCC (Ca_V_1) at pressure. The Ca_V_3.2 current reduction conforms to the above notion. However, in part of the studies (Grossman et al., 1991; Etzion and Grossman, 2000) Ca_V_1.2 involvement was excluded pharmacologically, while in others it was assumed absent based on information obtained from similar or identical preparations (Robitaille et al., 1990; French et al., 2002). In the chromaffin cells the channels were denoted as Ca_V_1 simply due to their similar behavior to these channels in neuronal cells, and have not been characterized pharmacologically. Lastly, the one change observed in Ca^2+^ currents in the bovine chromaffin cells was a slight increase at 40 MPa, which might support our finding of augmentation of currents in the Ca_V_1.2 (probably a different isoform) at lower pressure. Thus, the HP-induced increase of current in Ca_V_1.2 could be specific and excludes other types of channel of the same family (Ca_V_1).

The augmentation of the currents in Ca_V_1.2 channel at HP demonstrated here is reminiscent of another member of this protein superfamily behavior, the K^+^ channels. The non-inactivating “delayed rectifier” K^+^ channels pass greater steady-state currents at pressure in invertebrates such as squid (Henderson and Gilbert, 1975; Shrivastav et al., 1981; Conti et al., 1982a), snail (Harper et al., 1981), and lobster (Grossman and Kendig, 1984). Enhanced K^+^ currents at pressure were also proposed as the basis for the firing patterns of Ca^2+^ APs in guinea pig Purkinje cells (Etzion and Grossman, 1999), and for slowing of the sinus-node pacemaker activity in various mammalian hearts (Ornhagen and Hogan, 1977). On the other hand, the reduction of currents in the Ca_V_3.2 shown here is in agreement with other experiments concerned with an inactivating type of the K^+^ channel, demonstrating a depression of currents at pressure in snail (Harper et al., 1981) and in mouse *Shaker* B K^+^ channels expressed in *Xenopus* oocytes (Meyer and Heinemann, 1997; Schmalwasser et al., 1998).

Previous studies in our laboratory in rat dentate gyrus corticohippocampal slices suggested that high pressure increases the transfer function between synaptic inputs and somatic spike generation by granule cells, despite the observed reduction of single field excitatory post-synaptic potential (fEPSP) amplitude and slope by nearly 50% (Talpalar and Grossman, 2004). This suggests that high pressure depresses synaptic activity while increasing excitability in the neuronal dendrites but not in the axons. The Ca_V_1.2 channels that were studied here are known to be present in the cell bodies and proximal dendrites of neurons in the dentate gyrus (Hell et al., 1993). It is suggested that pressure-potentiated Ca_V_1.2 currents in the dendrite may boost subthreshold synaptic potentials to generate APs. Such a dendritic hyperexcitability could explain the increased transfer function and is a good example of another way through which pressure selective effects on VDCC might impact neuronal networks, other than synaptic transmission. Such an increased transfer function mechanism may conform to the hyperexcitability manifested in HPNS.

It is interesting to note that the Ca_V_1.2 is also expressed in the cardiac muscle, and the Ca_V_3.2 in the cardiac Purkinje fibers. Both changes in the maximal current amplitude described here correlate with previous findings: an increase in the contractility force of isolated rat hearts (Ornhagen and Sigurdsson, 1981; Gennser and Ornhagen, 1989a) and a decrease in the contraction pace in rat (Gennser and Ornhagen, 1989b) and humans (Linnarsson et al., 1999; Kurita et al., 2002).

A positive ionotropic pressure effect at steady-state that was reported for skeletal and cardiac muscles was previously explained by increase of cytosolic Ca^2+^ that may occur due to inhibition of Ca^2+^ removal from the cytosol, into either cellular or extracellular compartments (Daniels and Grossman, 2003). Our study may offer a different or additional mechanism for these changes in inotropy.

The augmented Ca_V_1.2 currents may increase neuronal dendritic excitability and therefore contribute to the generation of HPNS. The contribution to HPNS of the reduction of Ca_V_3.2 currents is less obvious. These low threshold activated channels are especially involved in generating bursting behavior and rhythmic activity in pace-maker neurons in the reticular thalamic nucleus, thalamus, striatum, and cortex, where they are involved in controlling sleep, awareness, executive function, movement planning and modulation, and sensory inputs, respectively. We may speculate that the current reduction will slow this activity and impair these neuronal “clocks” function. This may interfere with time processing and coincidence detection as well as motor and sensory functions in a manner similar to HPNS signs and symptoms. A slower rhythm at HP was indeed found in humans, where EEG waves shifted from α to θ patterns (Rostain et al., 1997). The same study also demonstrated sleep disturbances that were more prominent during the beginning of the compression, subsided later, and were abolished at decompression, suggesting both transient and reversible HP effect, similar to the results shown in our experiments.

The amplitude of slow after-hyperpolarization (sAHP) in rat CA1 was reduced by HP (Southan and Wann, 1996). This reduction could be explained by a depression of the SK potassium channel, responsible for the sAHP. But since this channel is activated by the rise of [Ca^2+^]_i_ during each AP, it can also be pointing to a reduction in Ca^2+^ influx through VDCCs, e.g., Ca_V_3.2, not only in the synaptic terminal but also along the axon and possibly in the soma and dendrites as well.

It is worth noting that our pressure effect on Ca_V_3.2 is saturating at about 1.1 MPa. This is well correlated with the pressure experienced by professional divers in the depth of seawater in which they begin to complain of the HPNS signs and symptoms. Therefore, this might be an indication that the Ca_V_3.2 is also involved in the underlying mechanism of this phenomenon. It is postulated that the mild symptoms observed at lower pressures such as impaired cognitive capabilities and motor dexterity are the consequence of pacemaker malfunction as a result of reduction in Ca_V_3.2 maximal currents.

#### Channels' conductance

Overall, the calculated conductance behavior relative to the membrane potential (Figure 4) is in accordance with the I-V curves described in Figure 2. Although generally the HP effect for Ca_V_1.2 and Ca_V_3.2 channels is opposite, at ΔV −10 mV both demonstrate an increase of conductance at HP (5.0 MPa, Figures 4C,D). The Ca_V_3.2 normalized-to-max conductance curve better shows the channels' postulated tendency to shift to an open state at HP in membrane potentials just over the threshold. However, once depolarization is increased the maximal currents and the conductance are depressed by HP. If HP raises the probability to shift to an open state, why would the currents depress? A possible explanation could be a stronger and faster inactivation of the channel at HP throughout the channels' activity range. Indeed, an indication for that can be seen by a decrease in both I_end_/I_max_ and Ʈ_Decay Fast_ at HP (Figures 5D,F and 8D,F, respectively).

The fact that only compression to 0.5 MPa caused a reduction in the normalized-to-max conductance curve above V_Imax_ in both channels (Figures 4E,F) may suggest a transient sensitivity of their “voltage sensor” to HP.

#### Currents TTP and Ʈ_Rise_

Both TTP and Ʈ_Rise_ elongate at HP for the Ca_V_1.2 channel, strongly suggesting a slower activation (Figures 6C, 7C, respectively). A similar HP effect was reported in previous studies on VDCCs in guinea pig single cerebellar Purkinje cells (probably Ca_V_2.1) (Etzion and Grossman, 1999) and frog motor nerve (possibly Ca_V_2.2) (Aviner et al., 2013). The velocity of an AP was also reduced at HP after a transient increase (Grossman and Kendig, 1986).

On the other hand, TTP was shortened in a narrow voltage range (−20 mV up to V_Imax_) in the Ca_V_3.2 (Figure 6D), suggesting a faster activation of the channel at HP. Nevertheless, shortening of TTP may also point to a faster inactivation process, which will make the maximal current appear earlier. This indeed was found in the Ca_V_3.2, where Ʈ_Decay Fast_ has shortened at HP (Figure 8D). Unfortunately, the inability to measure Ʈ_Rise_ for Ca_V_3.2 in this case prevents distinguishing the two options. However, the opposite HP effect on TTP, conductance, and maximal currents for these channels, together with the fact that Ʈ_Decay Fast_ was not significantly elongated for the Ca_V_1.2 at HP (Figure 8C) although TTP was increased, may indicate that for the Ca_V_3.2 Ʈ_Rise_ may be shortened at HP.

### Current inactivation

As expected for a fast inactivating channel, the Ca_V_3.2 shows a lower value of I_end_/I_max_ (stronger inactivation) than the Ca_V_1.2 for any given membrane potential (Figures 5A–D). At V_Imax_ the I_end_/I_max_ value has decreased for the Ca_V_1.2 at HP, and the same happened for the Ca_V_3.2 at ΔV −10 to −20 mV. This correlates well with smaller Ʈ_Decay Fast_ (faster inactivation) in both channels in this voltage range at HP (Figures 8C,D). On the other hand, stronger depolarization (ΔV 10–40 mV) at HP has led to weaker inactivation in the Ca_V_1.2 but not in the Ca_V_3.2 channel (Figures 5C,D). These findings are not supported by the measured Ʈ_Decay Fast_ in both channels in the same voltage range: it was not changed in the Ca_V_1.2 and it was still reduced in the Ca_V_3.2. For the latter it seems that since the inactivation has managed to reach its maximal values at the end of the 100 ms time window (end of depolarizing step), the faster Ʈ_Decay Fast_ at HP has only facilitated the already strong inactivation and did not change its values. For the Ca_V_1.2 channel the Ʈ_Decay Fast_ averages are generally higher at HP for ΔV 10 and above, although not statistically significant, but probably enough to weaken the measured inactivation. A slower inactivation at HP was also reported in Na^+^ channel in bovine chromaffin cells (Heinemann et al., 1987), but not in the *Helix* snail (Harper et al., 1981).

The flatter normalized-to-minimum curve of inactivation in Ca_V_1.2 at 0.1 MPa relative to HP curves (Figure 5E) and the linear behavior of Ʈ_Decay Fast_ at 0.1 MPa and after decompression as opposed to the behavior at HP (Figure 8C) both suggest that the molecular mechanism controlling the voltage-dependent inactivation is affected by HP.

Notwithstanding, no significant changes were observed in the Ʈ_Decay Slow_ at HP in both channels, supporting the concept of different mechanisms for the fast and slow inactivation (Hering et al., 2000; Sokolov et al., 2000), which can also react differently to external treatment (Livneh et al., 2006). It was also demonstrated that the molecular structures responsible for these two types of inactivation are differently located in the VDCC's protein (Berjukow et al., 1999) and that the fast inactivation may act similarly to the “ball and chain” mechanism in the K^+^ channel (Cens et al., 1999), while the slow inactivation seems to be at least partially dependent on the interaction between α_1_ and β subunits (Sokolov et al., 2000). We suggest that the selective HP effect between the two channels' inactivation (I_end_/I_max_ and Ʈ_Decay_) may arise from different conformational changes at HP, resulting from a different basic spatial organization of the channels, specifically their inactivation controlling regions.

### Currents deactivation

#### Currents Ʈ_Tail_

Compression to 0.5 and 5.0 MPa, but not 2.5 MPa, shortened Ʈ_Tail_ for Ca_V_1.2, implying a faster deactivation of the channel at HP (Figure 10B). The lack of consistency in the HP effect on Ʈ_Tail_ may suggest a transient effect caused by the initial compression (0.5 MPa), and the inability of the deactivating mechanism to compensate for the higher HP (5.0 MPa).

The normalized curves show that in the Ca_V_1.2 the reduction of Ʈ_Tail_ has saturated at V_Imax_ at normobaric and HP (Figure 10C). However, at 5.0 MPa saturation was achieved already at ΔV −10 mV, which may indicate that the closure of the channel is facilitated by high HP.

## Summary

HP affected the behavior of both Ca_V_1.2 and Ca_V_3.2, whether throughout their membrane potential activity range (maximal current, conductance, Ʈ_Tail_ at 5.0 MPa), or just at a confined voltage range (Ʈ_Rise_, TTP, inactivation, Ʈ_Decay Fast_). The HP effect on the two channels was generally opposite (maximal currents, conductance, TTP), but some kinetic traits shared the same HP-induced tendencies (inactivation at V_Imax_, Ʈ_Decay Fast_). Some of the effects may indicate a transient nature (conductance and partially Ʈ_Tail_), while other suggested that the HP effect can be reversible (mostly for Ca_V_1.2; Ʈ_Decay Fast_, Ʈ_Tail_, and partially also maximal current and Ʈ_Rise_). A summary of the major HP-induced findings is given in Table 1.

**Table 1:** **General qualitative effect of HP found in our study on channel characteristics**.

	**I_max_**	**Conductance**	**Inactivation**	**TTP**	**Ʈ_Rise_**	**Ʈ_Decay Fast_**	**Ʈ_Decay Slow_**	**Ʈ_Tail_**
Ca_V_1.2	↑	↑	↑ (↓)	↑	↑	↓ (=)	=	↓
Ca_V_3.2	↓	↓	↑(=)	↓	N.A.	↓	= (↓)	N.A.

*↑, increase; ↓, decrease; =, no change; ( ), stronger depolarization*.

These changes in the response to depolarization, in both magnitude and kinetics, would undoubtedly influence these channels' functionality in neurons. Decrements in locomotor activity, myoclonus, tremors, changes in EEG, and sleep disorders, all part of the HPNS phenomenon, may be the manifestation of these HP-induced changes.

### General considerations

The effect of HP can be targeted either at the channel (and its subunits) or at any external modulator. Some of the characteristics examined in this study exhibited sensitivity (sometimes opposite) to HP that was also dependent on the membrane potential, suggesting that HP affects the channel itself. More specifically, HP may target the channels' “voltage sensor” and thus affect voltage-dependent mechanisms of activation, inactivation, and deactivation.

Several studies have demonstrated that replacing a section within a subunit (Tang et al., 1993; Zhang et al., 1994) or even a single amino acid (Bourinet et al., 1999; Hans et al., 1999) can dramatically change its reaction to depolarization. Furthermore, it was suggested that these changes are caused by different spatial organization of the subunits, influencing their interactions (Hering et al., 2000).

Similarly, the HP-induced effects demonstrated in this study may indicate that the conformational changes involved in the channels' activity are facilitated (e.g., activation in the Ca_V_1.2, fast inactivation in the Ca_V_3.2) or opposed (e.g., activation in the Ca_V_3.2, rate of activation in the Ca_V_1.2) by an elevated ambient pressure. Indeed, this notion is supported by the calculated activation volumes corresponding to these processes, probably affecting the total ionic flux through the channels at HP. Furthermore, even the basic (i.e., not activated) spatial organization of the subunits and their interactions may be altered by the HP application (e.g., a segment within S4 transmembrane region, holding the positively charged amino acids sequence that serve as a voltage sensor), thus leading to the HP sensitivity shown here. For example, the human and rat glycine receptor affinity to its ligand was reduced at HP (Roberts et al., 1996). The reduced activity of this inhibitory mechanism may explain the tendency to hyperexcitability upon exposure to HP (HPNS manifestations). A genetic sequencing of the receptor demonstrated a few amino acid switches between the mammals mentioned above and the *Pilot whale*, one of them in the ligand binding site from the non-polar cysteine (human and rat) to the polar arginine (C41R). This switch may protect the whale from hyperexcitability at HP and provide it with the ability to dive to extreme depths.

It can therefore be postulated that performing HP experiments on a specific VDCC with altered subunits or a VDCC chimera might further elucidate the HP targeted site. Preliminary results from experiments on the NMDA receptor at HP may suggest that even changes in the extracellular N terminal domain can influence the magnitude and direction of the pressure effect (Mor et al., 2012).

## Conclusions

- HP Modulation of various VDCCs is part of HPNS mechanisms and may explain some of its signs and symptoms.
- HP effects could be quite selective for the type of channel and/or various mechanisms underlying the channels' activity.
- HP effects on the channels' kinetics should be extensively studied in order to reveal small functional differences under HP conditions.
- Our study emphasizes the importance of pressure modulation of the membrane potential “sensor,” which may determine the extent of pressure effects on the channels.

### Conflict of interest statement

The authors declare that the research was conducted in the absence of any commercial or financial relationships that could be construed as a potential conflict of interest.
